# Dysregulated Anaerobic Glycolysis in Podocytes is Relevant to the Progression of Focal Segmental Glomerulosclerosis

**DOI:** 10.1016/j.ekir.2025.06.022

**Published:** 2025-06-18

**Authors:** Masahiro Sugimura, Kayaho Maeda, Katsuaki Shibata, Hiroshi Seko, Yohei Kozaki, Akiyoshi Hirayama, Tomoyoshi Soga, Takaya Ozeki, Yuka Sato, Noritoshi Kato, Tomoki Kosugi, Kenji Kadomatsu, Shoichi Maruyama

**Affiliations:** 1 Department of Nephrology, Nagoya University Graduate School of Medicine, Nagoya, Japan; 2 Department of Biochemistry, Nagoya University Graduate School of Medicine, Nagoya, Japan; 3 Institute for Glyco-core Research, Nagoya University, Nagoya, Japan; 4 Institute for Advanced Biosciences, Keio University, Tsuruoka, Japan

**Keywords:** anaerobic glycolysis, corticosteroid resistance, energy metabolism, focal segmental glomerulosclerosis, podocyte

## Abstract

**Introduction:**

Minimal change disease (MCD) and focal segmental glomerulosclerosis (FSGS) are podocytopathies with varying clinical courses and therapeutic responses. FSGS often leads to end-stage renal disease. Consequently, their heterogeneity requires case stratification and pathophysiological elucidation. The involvement of energy metabolism in FSGS pathogenesis and stratification has not been clarified. Therefore, this study aimed to verify whether evaluating energy kinetics can be a new approach to MCD or FSGS stratification and explore the role of energy metabolism in MCD or FSGS.

**Methods:**

Cultured human podocytes were treated with sera from patients with biopsy-confirmed MCD or FSGS. Serum-treated podocytes were analyzed for apoptosis using flow cytometry, metabolomics via mass spectrometry, and real-time adenosine triphosphate (ATP) production rates using an extracellular flux analyzer. Adriamycin-induced nephropathy was induced in podocyte-specific lactate dehydrogenase (LDH) A (LDHA)-deficient and control mice.

**Results:**

The sera from patients with FSGS significantly induced apoptosis in human podocytes compared with those from individuals with MCD. Apoptosis severity was associated with segmental obliteration and corticosteroid resistance. Metabolomic analysis revealed differences in anaerobic glycolysis and tricarboxylic acid cycle (TCA)-related metabolites in podocytes exposed to the sera of patients with MCD and FSGS. In the podocytes treated with sera from patients with FSGS, glycolytic ATP production significantly decreased in cases with high apoptosis rates. The sera from patients with FSGS suppressed LDHA activity, suppressed α-actinin 4 (ACTN4) expression, and promoted actin remodeling of podocytes. Segmental sclerosis was more prominent in podocyte-specific LDHA-deficient mice with adriamycin-induced nephropathy than in control mice.

**Conclusion:**

FSGS progression was associated with decreased anaerobic glycolysis in podocytes.

## Graphical abstract

**Figure:**
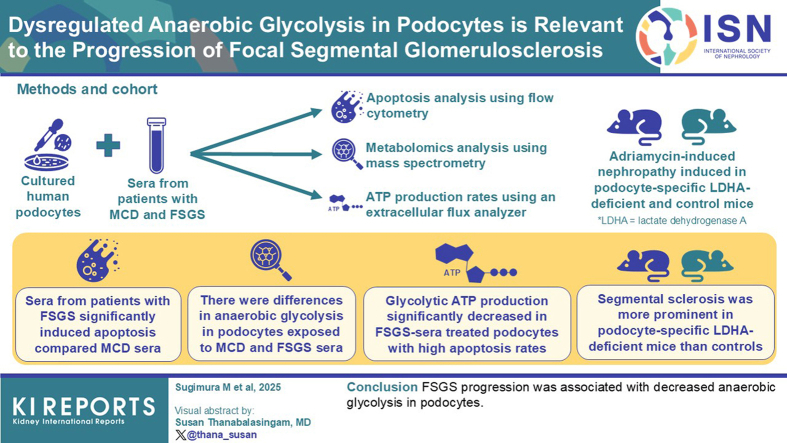

MCD and FSGS represent podocytopathies with diverse clinical courses and treatment responses.^1^^,^^2^ Primary FSGS involving circulating factors is a major cause of corticosteroid-resistant nephrotic syndrome (SRNS) and often leads to end-stage renal disease. Because FSGS lesions develop from various causes, stratifying patients and characterizing each population properly is important.^3^ The conventional classification of FSGS, based on clinical symptoms and pathological findings, is insufficient for predicting prognosis or identifying pathophysiology.^4^, ^5^, ^6^ Identifying disease entities associated with the poor clinical prognosis of MCD or FSGS using novel approaches is necessary.

Podocytes are highly differentiated cells that form a slit membrane and regulate the glomerular filtration barrier permeability. Disruption of this structure results in glomerular barrier function loss, leading to proteinuria. The kidney is a high-energy organ susceptible to injury when abnormal energy metabolism occurs, particularly in the renal tubules.^7^, ^8^, ^9^ Podocytes require a constant energy supply to maintain their complex structure; disrupted ATP production can lead to irreversible cell death.^10^

Recent studies have indicated that glycolysis is crucial in podocyte differentiation, where anaerobic glycolysis is the primary energy source under physiological conditions.^11^^,^^12^ Understanding the function of glycolysis in injured podocytes is important. Mutations in mitochondria-related genes, such as coenzyme Q10 synthesis-related enzymes, cause hereditary FSGS.^13^ Patients with corticosteroid-resistant FSGS exhibit higher levels of reactive oxygen species in isolated glomeruli than those with corticosteroid-sensitive nephrotic syndrome (SSNS), indicating mitochondrial dysfunction involvement.^14^ However, the role of energy metabolism in primary FSGS remains unclear.

Therefore, this study verified whether evaluating energy kinetics can be a new approach to MCD or FSGS stratification and explored the role of energy metabolism in MCD or FSGS.

## Methods

### Human Samples

Serum samples were obtained from patients with MCD and FSGS diagnosed using kidney biopsy, and healthy individuals from the Nagoya Kidney Disease Registry between 2014 and 2018. These samples were obtained from patients with FSGS with complete remission posttreatment. The Institutional Review Board on Human Subjects of the Nagoya University Hospital approved the protocol for obtaining the biopsy samples from patients with MCD and FSGS (approval number: 1135). This study was conducted following the Declaration of Helsinki guidelines. Written informed consent was obtained from all patients before the kidney biopsy. The sera from patients were obtained during kidney biopsy. Clinical data for all patients with MCD and FSGS were monitored for 2 years after the initial treatment.

### Animal Studies

Podocyte-specific LDHA-deficient mice (*Ldha*^fl/fl^/Cre^+^) were generated by crossing *Ldha*-deficient mice (Jackson Laboratory; strain 030112) with B6.Cg-Tg (*NPHS2*-cre) 295Lbh/J mice (Jackson Laboratory; strain 008205). Homozygous mice lacking Cre expression (*Ldha*^fl/fl^/Cre^−^) were used as controls. A single i.v. injection of adriamycin (17 mg/kg body weight) was administered to 8- to 10-week-old male mice. Urine samples were obtained after 1, 2, and 4 weeks, and the mice were euthanized 4 weeks later. The urinary albumin-to-creatinine ratio was measured using an AlbuwellM and creatinine companion (Exocell, PA).

### Cell Culture and Treatments

The immortalized human podocyte cell line was cultured as previously described.^15^ Podocytes were cultured in RPMI-1640 with 10% fetal bovine serum, insulin-transferrin-selenium, 100 U/ml penicillin G, and 100 μg/ml streptomycin for proliferation. They proliferated at 33 °C and differentiated into mature podocytes in 10 to 14 days after being transferred to 37 °C because of the temperature-sensitive SV-40T gene and a telomerase gene. Differentiated podocytes were treated with recombinant human tumor necrosis factor (100 ng/ml; R&D Systems, MN), adriamycin (0.25 μg/ml; Selleck, TX), FX11 (1–10 μM; Sigma-Aldrich, MO), and 10% sera from patients. The cells were harvested using a cell scraper without trypsinization.

### Immunohistochemistry

Formalin-fixed tissues from *Ldha*^fl/fl^/Cre^+^ and *Ldha*^fl/fl^/Cre^−^ mice with adriamycin-induced nephropathy were used for periodic acid-Schiff and indirect immunoperoxidase staining, as previously described.^16^ The tissue sections were deparaffinized with xylene and rehydrated in a graded ethanol series. Endogenous peroxidase activity was blocked using 3% hydrogen peroxide, and antigen was retrieved using ethylenediaminetetraacetic acid. After blocking, the sections were incubated overnight at 4 °C with the primary antibody, Wilms tumor 1 (Abcam, Cambridge, UK). Each section was stained using indirect peroxidase staining, and the target antigen was visualized using 3,3'-diaminobenzidine, followed by counterstaining with hematoxylin. Segmental sclerosis frequency was determined by calculating the affected glomeruli proportion in the periodic acid-Schiff–stained sections. Wilms tumor 1–positive cells were counted as podocyte numbers in all glomeruli per section. Kidney samples were scored in a blind manner by a nephropathologist.

### Transfection of Small, Interfering RNA

Human podocytes were cultured and differentiated for 7 to 10 days, followed by transfection with LDHA#1 (small, interfering RNA [siRNA] ID, s350), LDHA#2 (siRNA ID, s351), or control small-interfering RNAs (siRNAs) using Lipofectamine RNAiMAX Transfection Reagent (Thermo Fisher Scientific, MA) per the manufacturer’s protocols. After 72 hours of culture, the cells were collected for experimentation.

### Seahorse Metabolic Analyzer Assays

Podocytes were grown on collagen 1–coated XFp cell culture plates in the RPMI-1640 medium. Cells were seeded at a density of 20,000 cells/well. One day before the assay, the XFp cartridge plate was hydrated overnight with H_2_O and replaced with a calibrant for 1 hour in a non-CO_2_ incubator. The cell plates were washed 3 times, replaced with warmed Seahorse XF RPMI medium (pH 7.4) and equilibrated at 37 °C in a non-CO_2_ incubator for 45 to 60 minutes. Next, the tubes were replaced immediately before the assay. Assay conditions and setup were performed using Seahorse XFp Analyzer with XFp real-time ATP rate Assay. XFp Glycolysis Stress Test (Agilent Technologies, CA) was performed following the manufacturer’s instructions. All Seahorse assay data were analyzed using Seahorse Wave v2.6.1 Software (Agilent Technologies, CA).

### Capillary Electrophoresis–Time-Of-Flight Mass Spectrometry–Based Untargeted Metabolomic Experiments

Metabolites were extracted from serum-treated podocytes as previously described.^17^ The resulting solutions were transferred to 5 kDa cutoff centrifugal filter tubes for capillary electrophoresis–time-of-flight mass spectrometry (Agilent Technologies, CA) analysis.^18^ Furthermore, the resulting data were analyzed with MetaboAnalyst version 5.0 (www. metaboanalyst. ca).

### Flow Cytometry

Apoptosis was analyzed using flow cytometry following the manufacturer’s instructions (APC Annexin V Apoptosis Detection Kit with 7-AAD; BioLegend Inc, CA). All flow cytometry data were acquired on a BD Canto II (Becton Dickinson and Company, NJ) and analyzed using the FlowJo software version 10.6.1 (Tree Star, OR).

### Phalloidin Staining

Podocytes were seeded on type I collagen 4-well culture slides (Corning, NY), washed 2 times with phosphate-buffered saline (PBS), and fixed in 4% paraformaldehyde for 15 minutes. They were permeabilized with 0.1% Triton X-100 in PBS for 15 minutes and blocked with PBS containing 5% bovine serum albumin for 30 minutes. Subsequently, the cells were stained with AF488-labeled phalloidin (Thermo Fisher Scientific, MA) for 1 hour at room temperature, washed 3 times with PBS, and mounted on glass slides with a mounting medium containing 4′,6-diamidino-2-phenylindole. Stained specimens were analyzed using a BZ9000 fluorescence microscope. Actin fiber consistency was measured using the Orientation J plugin in ImageJ.^19^

### Wound Healing Assay

This was performed as previously described.^20^ Podocytes were seeded in type 1 collagen-coated 6-well plates. Each well was wound with a sterile 200 μl micropipette, washed with PBS, and replaced with fresh medium. The wounds were imaged with a phase-contrast microscope after 12 hours, and the wound area was measured using the wound healing size tool plugin for ImageJ.^21^

### Real-Time Polymerase Chain Reaction

Total RNA was extracted from the isolated glomeruli using the RNeasy Micro Kit (QIAGEN, Hilden, Germany). Real-time polymerase chain reaction was performed using Applied Biosystems StepOnePlus Real-Time polymerase chain reaction system and TaqMan Gene Expression Assay (Applied Biosystems, MA). Gene expression data were normalized to *Actb* encoding β-actin. The TaqMan probes and primers used were *Hk1* (Mm00439344_m1), *Gpi1* (Mm01962484_u1), *Pfkm* (Mm01309576_m1), *Gapdh* (Mm99999915_g1), *Pgk1* (Mm00435617_m1), *Pkm* (Mm00834102_gH), *Pklr* (Mm00443090_m1), *Ldha* (Mm01612132_g1), *Actb* (Mm02619580_g1)*, ACTN4* (Hs00245168_m1), *CD2AP* (Hs00961458_m1), *NPHS1* (Hs00190446_m1), *SYNPO* (Hs00702468_s1), and *PPIA* (Hs04194521_s1). The Applied Biosystems Sequence Detection software version 1.3.1 (Applied Biosystems) was used for analysis.

### Western Blotting

Total protein was extracted from podocytes in radioimmunoprecipitation assay buffer supplemented with protease and phosphatase inhibitors (Thermo Fisher Scientific, MA). Protein concentration was quantified using the bicinchoninic acid protein assay (Thermo Fisher Scientific, MA). Next, protein samples were separated on 5% to 12% polyacrylamide gels and transferred to a polyvinylidene difluoride membrane. The membranes were blocked with 5% nonfat dry milk for 1 hour at room temperature and incubated overnight at 4 °C with antibodies against actinin 4 (Abcam, Cambridge, UK), LDHA, phosphorylated mechanistic target of rapamycin (mTOR), mTOR, and β-actin (Cell Signaling Technology, MA). Horseradish peroxidase-linked goat antirabbit/mouse IgG antibody was used as the secondary antibody for 1 hour at room temperature. The bands and blots were visualized and analyzed using an ECL system (Amersham Imager 680; Cytiva, MA).

### Lactate Dehydrogenase Activity Assay

Serum-treated podocytes were used for the LDHA enzyme activity assay employing the LDHA Activity Assay Kit, following the manufacturer’s instructions, which is based on the fact that LDH reduces NAD to NADH and can be detected using colorimetry (450 nm). The cells were homogenized on ice in an LDH assay buffer. Samples were incubated in 96-well plates with the LDH substrate mix and detected at 450 nm using a microplate reader (Multiskan Sky; Thermo Fisher Scientific, MA). FX11- and siRNA-treated podocytes were measured similarly.

### Lactate Production Assay

This was performed as previously described.^18^ Lactate production in the patient sera-treated medium was measured after 24 hours using a lactate test meter (ARKRAY, Kyoto, Japan).

### Isolation of Glomeruli

Mouse glomeruli were harvested using a magnetic separation protocol as previously described.^19^ Briefly, mice perfused with Dynabeads M-450 Tosylactivated (Thermo Fisher Scientific, MA) were euthanized. The kidneys were minced, digested with type 1 collagenase (Worthington Biochemical, NJ) and DNase 1 (Roche, Basel, Switzerland) for 30 minutes at 37 °C, and passed through a 100 μm filter. Glomeruli captured using Dynabeads were magnetically separated and washed with PBS.

### Statistical Analyses

All statistical analyses were performed using GraphPad Prism version 10.0 software. Statistical significance was determined using 2-tailed *t* tests or Mann-Whitney U tests for between-groups comparison and 1-way analysis of variance with Tukey’s multiple comparison tests for ≥ 3 groups. Pearson correlation was used to calculate the correlation coefficient *r.* Statistical significance was set at *P* < 0.05.

## Results

### Sera From Patients With FSGS/SRNS–Induced Podocyte Injury

**Figure 1:**
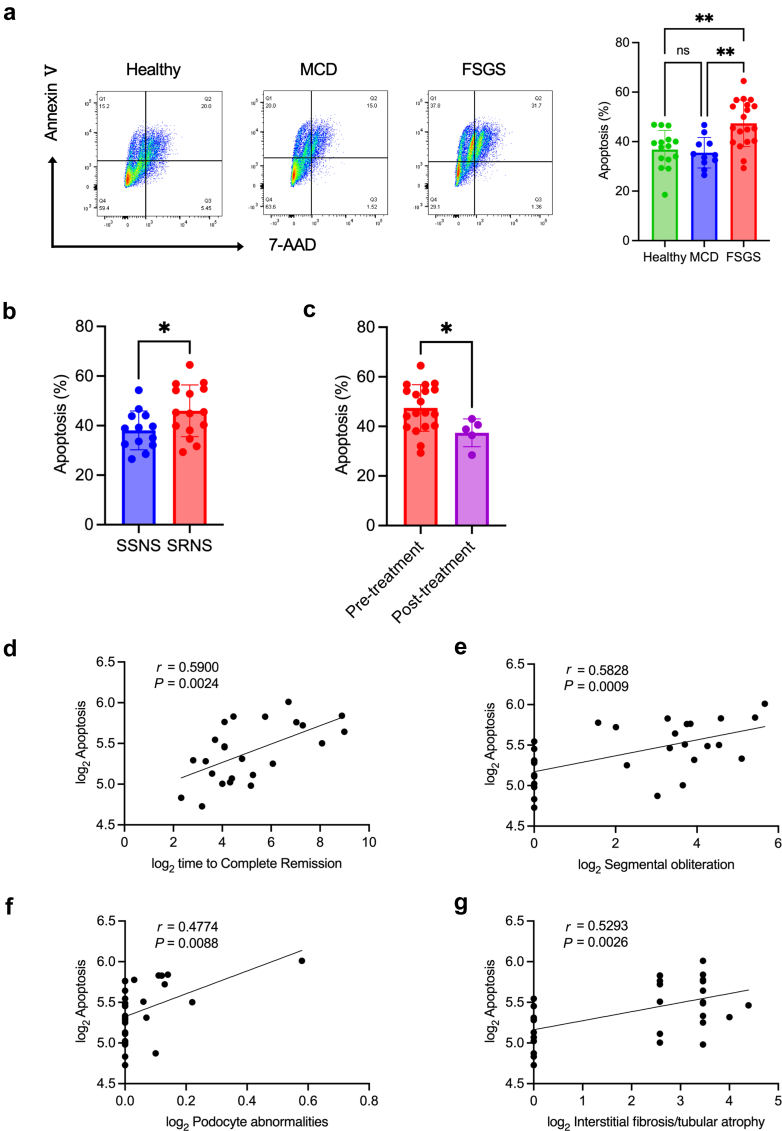
Sera from patients with FSGS/SRNS induce podocyte injury, which correlates with time to complete remission and pathological score. (a) Apoptosis rate of serum-treated podocytes from healthy individuals (*n* = 15) and patients with MCD (*n* = 11) or FSGS (*n* = 19) detected by flow cytometry. ∗∗*P* < 0.01. (b) Apoptosis rate of serum-treated podocytes from patients with SSNS (*n* = 13) or SRNS (*n* = 15) detected by flow cytometry. ∗*P* < 0.05. (c) Apoptosis rate of serum-treated podocytes from patients with pretreatment (*n* = 19) or posttreatment (*n* = 5) FSGS detected by flow cytometry. ∗*P* < 0.05. (d–g) Scatterplot showing correlations between podocyte apoptosis and (d) time to complete remission, (e) segmental obliteration, (f) podocyte abnormalities, and (g) interstitial fibrosis and tubular atrophy. FSGS, focal segmental glomerulosclerosis; MCD, minimal change disease; SRNS, corticosteroid-resistant nephrotic syndrome; SSNS, corticosteroid-sensitive nephrotic syndrome.

We treated cultured human podocytes with sera from healthy individuals (*n* = 15) and patients with kidney biopsy-proven MCD (*n* = 11) and primary FSGS (*n* = 19) and assessed podocyte apoptosis after 48 hours (Table 1). The sera-treated podocytes from patients with FSGS showed a significant increase in apoptosis compared with those from healthy individuals and patients with MCD (Figure 1a). In addition, the patients were categorized into the SSNS (with complete remission) and SRNS (without complete remission after 4 weeks of standard corticosteroid therapy) groups. Sera-treated podocytes from patients with SRNS versus SSNS significantly increased apoptosis (Figure 1b). The sera from patients with FSGS with complete remission posttreatment showed reduced apoptosis induction compared with pretreatment sera (Figure 1c). Apoptosis rate was positively correlated with days to complete remission or pathological findings, including segmental obliteration, podocyte abnormalities, and interstitial fibrosis and tubular atrophy (Figure 1d–g). Therefore, the sera from patients with FSGS/SRNS induced podocyte injury and assessing the extent of damage enabled the identification of corticosteroid resistance or cases with poor prognostic pathological findings.

**Table 1:** Clinical characteristics

Characteristics	MCD (*n* = 11)	FSGS (*n* = 19)	*P*-value
Age, mean ± SD (yrs)	33.6 ± 11.1	52.6 ± 14.4	0.001
Sex (%)	55%	58%	0.864
Creatinine, mean ± SD (mg/dl)	1.37 ± 1.92	1.44 ± 1.04	0.909
Albumin, mean ± SD (g/dl)	1.49 ± 0.66	1.95 ± 0.70	0.098
Proteinuria, mean ± SD (g/gCr)	13.87 ± 9.54	8.65 ± 4.85	0.065
HbA1c, mean ± SD (%)	5.43 ± 0.31	5.83 ± 0.56	0.067
Immunosuppressive treatment at the time of kidney biopsy (%)	55%	26%	0.131
Complete remission rate (%)	100%	68%	0.037
Days to remission, mean ± SD (d)	17.09 ± 10.55	143.54 ± 164.15	0.023
Corticosteroid sensitivity (%)	82%	24%	0.002

FSGS, focal segmental glomerulosclerosis; HbA1c, glycated hemoglobin; MCD, minimal change disease.

### TCA Cycle and Glycolytic Metabolites Were Altered in Corticosteroid-Resistant FSGS Sera-Treated Podocytes

**Figure 2:**
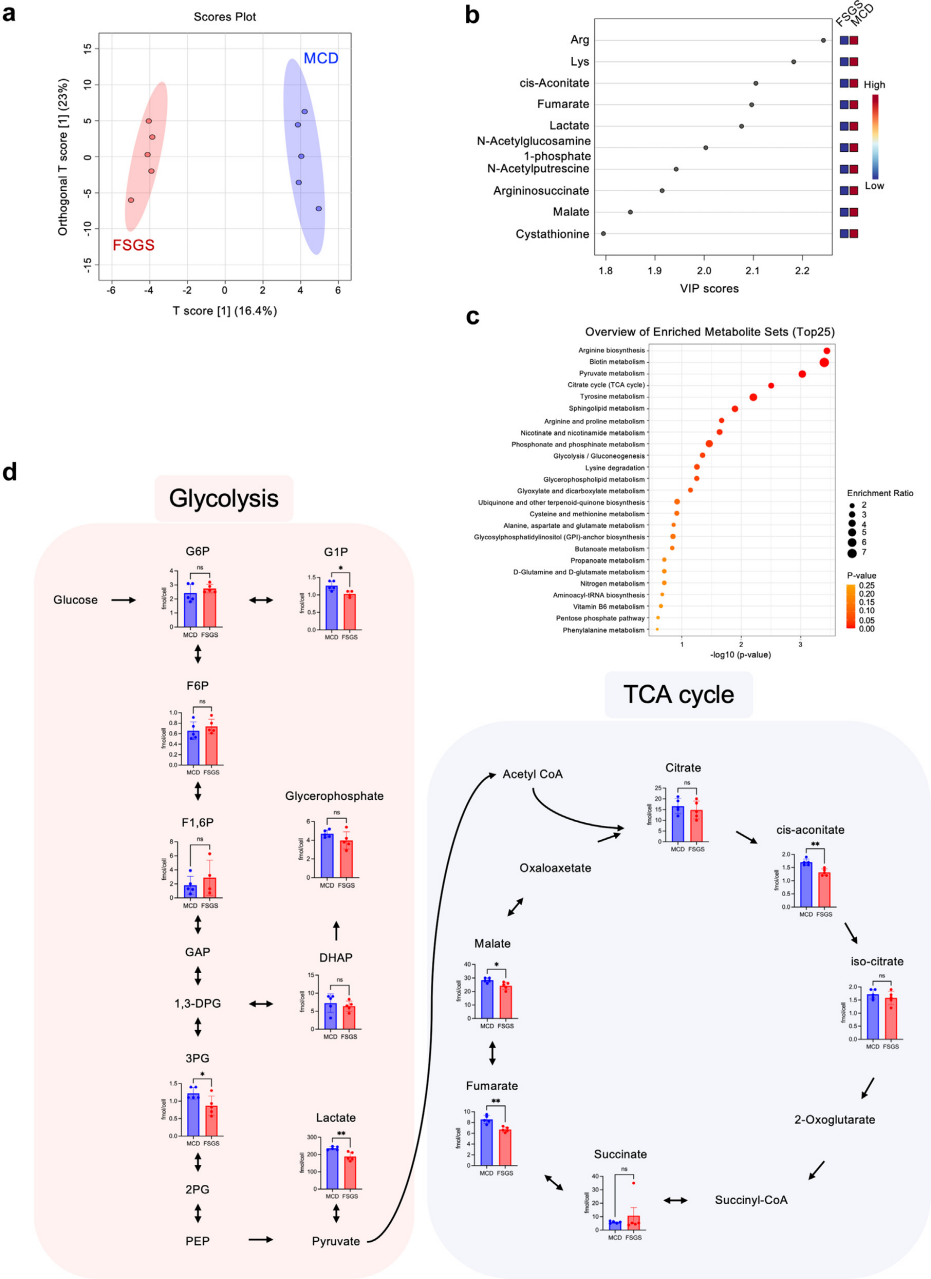
Podocytes treated with sera from patients with corticosteroid-resistant FSGS exhibit different metabolic profiles compared with those with MCD. (a) OPLS-DA of metabolites of serum-treated podocytes from patients with MCD or FSGS (*n* = 5 each). (b) Top 10 of VIP score plot of metabolites of serum-treated podocytes from patients with MCD or FSGS. (c) Enrichment analysis of metabolites of serum-treated podocytes from patients with MCD or FSGS. (d) Pathways of glycolytic metabolites of serum-treated podocytes from patients with MCD or FSGS. FSGS, focal segmental glomerulosclerosis; MCD, minimal change disease; OPLS-DA, orthogonal partial least squares discriminant analysis; VIP, variable importance in projection.

We conducted a metabolomic analysis of the sera-treated podocytes from patients with MCD and corticosteroid-resistant FSGS to investigate the relationship between serum-induced podocyte injury and energy metabolism. An overview of the dataset using orthogonal partial least squares discriminant analysis showed clear clustering and separation between patients with MCD and FSGS (Figure 2a). TCA and glycolytic metabolites were in the top 10 of the Variable Importance in Projection scores, and the sera-treated podocytes from patients with FSGS versus MCD had lower values of these metabolites (Figure 2b). Enrichment analysis also showed that the pyruvate metabolism, TCA cycle, and glycolysis are high in the enriched metabolite set (Figure 2c). Comparison of each glycolytic metabolite showed that glucose-1-phosphate, 3-phosphoglycerate, and lactate levels in serum-treated podocytes from patients with FSGS versus MCD were significantly lower (Figure 2d, Supplementary Figure S1). For each metabolite of the TCA cycle, malate and fumarate levels in the serum-treated podocytes from patients with FSGS versus MCD were significantly lower (Figure 2d).

### Podocyte Injury Inversely Correlated With Glycolytic ATP Production Rate but not With the Total or Mitochondrial ATP Production Rate

**Figure 3:**
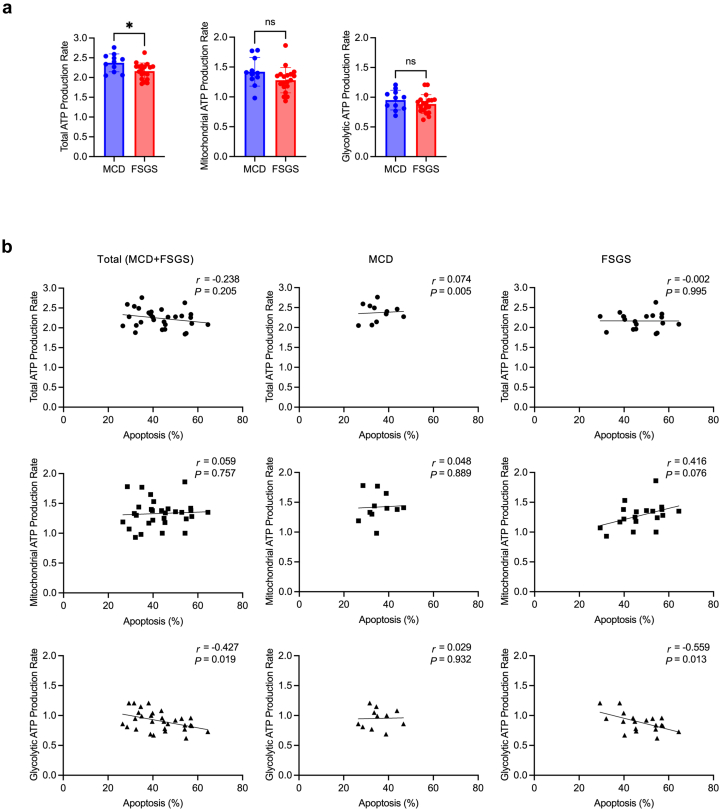
Podocyte injury inversely correlates with glycolytic ATP but not with total or mitochondrial ATP production rate in podocytes treated with sera from patients with FSGS. (a) Real-time ATP rate assay of serum-treated podocytes from patients with MCD (*n* = 11) or FSGS (*n* = 19) detected by extracellular flux analyzer. (b) Scatterplot showing the correlation between podocyte apoptosis and total/mitochondrial/glycolytic ATP production rate (MCD + FSGS [*n* = 30], MCD [*n* = 11], and FSGS [*n* = 19]). ATP, adenosine triphosphate; FSGS, focal segmental glomerulosclerosis; MCD, minimal change disease.

Metabolic changes in serum-treated podocytes were evaluated using Seahorse extracellular flux analyzer to further investigate the roles of glycolysis and mitochondrial respiration in serum-mediated podocyte injury. In real-time ATP rate Assay, serum-treated podocytes from patients with MCD versus FSGS showed a significant increase in total ATP production rates, although mitochondrial and glycolytic ATP production rates did not differ (Figure 3a). The SRNS and SSNS analyses showed no significant differences between the 2 groups (Supplementary Figure S2a). Furthermore, the correlation between ATP production rate and apoptosis was analyzed individually (Figure 3b), with no significant correlation found between total or mitochondrial ATP production rate and apoptosis in sera-treated podocytes from patients with MCD or FSGS. However, a significant inverse correlation was found between the glycolytic ATP production rate and apoptosis in all patients, as well as in the sera-treated podocytes from patients with FSGS. A significant inverse relationship was found between apoptosis and glycolytic ATP production rate in the sera of patients with SRNS but not in those of individuals with SSNS (Supplementary Figure S2b). Considering these results, we focused on glycolysis and performed a glycolysis stress test. No significant differences were found in glycolysis, glycolytic capacity, or glycolytic reserve between the sera from patients with MCD or FSGS (Supplementary Figure S3a). The degree of apoptosis in the sera of patients with MCD and FSGS was inversely correlated with glycolytic capacity (Supplementary Figure S3b).

### Anaerobic Glycolysis was Suppressed in Sera-Treated Podocytes From Patients With FSGS and Adriamycin-Induced Nephropathy

**Figure 4:**
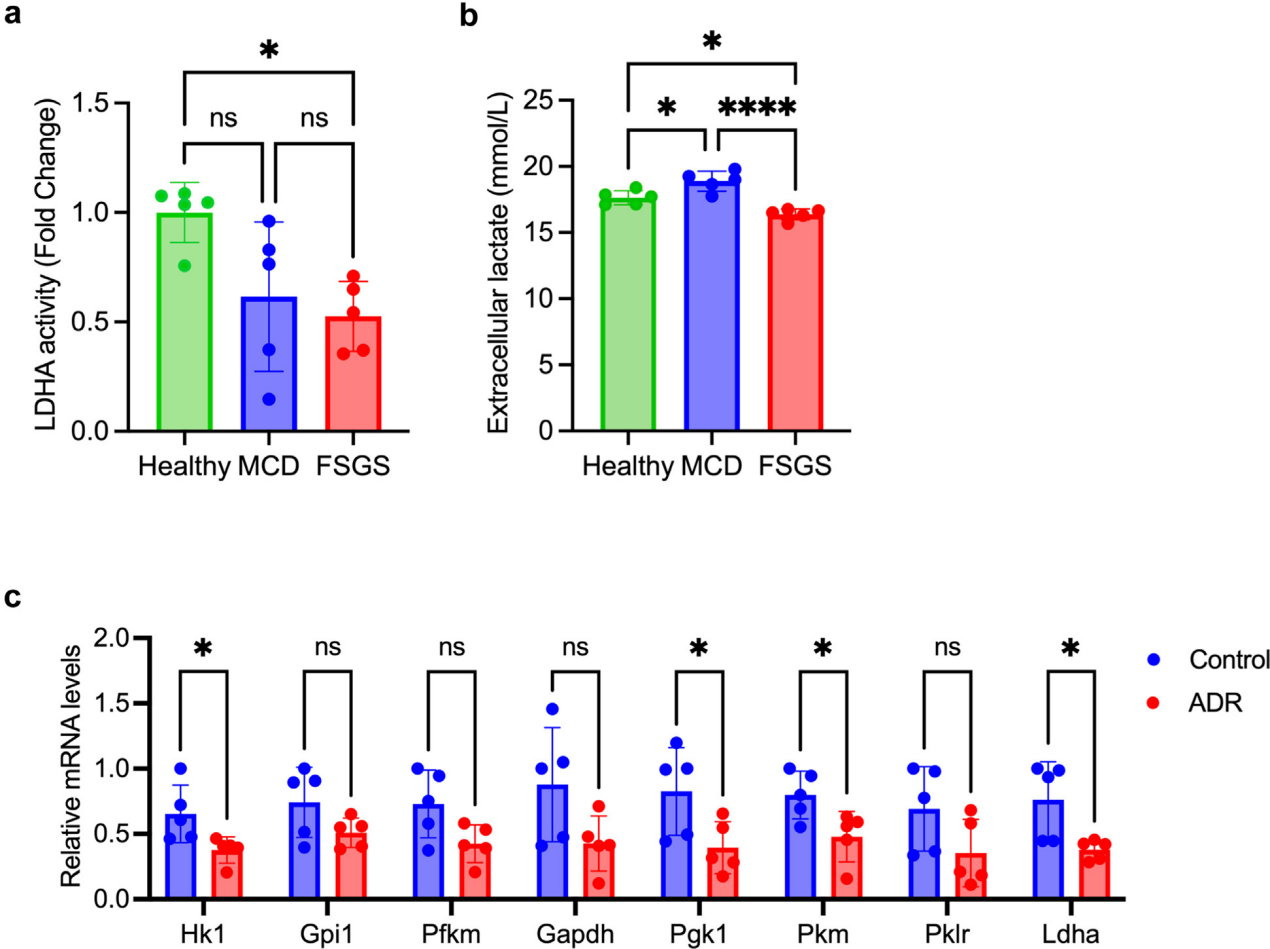
Podocyte injury is associated with reduced glycolysis, particularly in LDHA activity. (a) LDHA activity of serum-treated podocytes from healthy individuals and patients with MCD or FSGS (*n* = 5 each). ∗*P* < 0.05. (b) Extracellular lactate levels in sera-treated podocytes from healthy individuals and patients with MCD or FSGS (*n* = 5 each). ∗*P* < 0.05, ∗∗∗∗*P* < 0.0001. (c) Glycolytic enzymes of glomeruli in control and adriamycin-induced nephropathy groups. (*n* = 5). ∗*P* < 0.05. FSGS, focal segmental glomerulosclerosis; LDHA, lactate dehydrogenase A; MCD, minimal change disease.

In the metabolomic analysis, lactate levels were significantly decreased in sera-treated podocytes from patients with corticosteroid-resistant FSGS (Figure 2d). To further investigate the relationship between anaerobic glycolysis and FSGS, LDHA activity was assessed in the serum-treated podocytes from healthy individuals and patients with MCD and FSGS. Its activity was significantly reduced in the sera-treated podocytes from patients with FSGS versus those from healthy individuals (Figure 4a). The extracellular lactate levels were lower in the sera-treated cultures from patients with FSGS than in those from healthy individuals or patients with MCD (Figure 4b). We used the FSGS mouse model of adriamycin-induced nephropathy to evaluate overall glycolysis during podocyte injury *in vivo*. Glycolytic enzyme expression in the glomeruli isolated from mice with adriamycin-induced nephropathy was measured. The glomeruli in adriamycin-induced nephropathy showed significant reductions in glycolytic enzymes *Hk1*, *Pgk1*, *Pkm*, and *Ldha* compared with the control glomeruli (Figure 4c).

### LDHA Inhibition Contributed to Podocyte Injury

**Figure 5:**
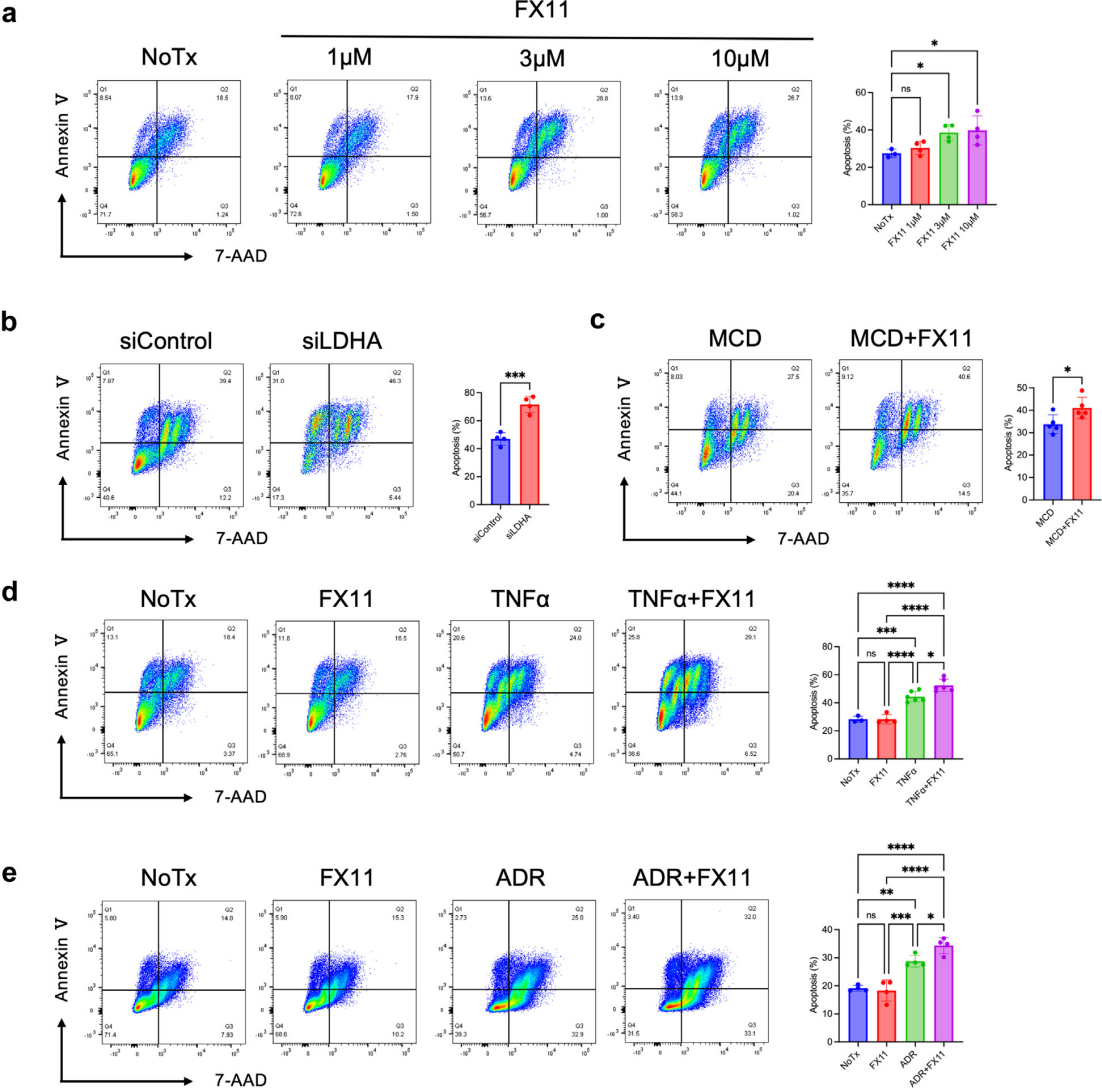
LDHA inhibition contributes to podocyte injury *in vitro*. (a) Apoptosis rate of podocytes detected by flow cytometry with or without FX11 (LDHA inhibitor) treatments (1–10 μM) (*n* = 5). (b) Apoptosis rate of podocytes with or without LDHA silencing detected by flow cytometry (*n* = 5). (c) Apoptosis rate of serum-treated podocytes from patients with MCD with or without low concentration FX11 (1 μM) administration detected by flow cytometry (*n* = 5). (d, e) Apoptosis rate of podocytes detected by flow cytometry in different groups with TNF-α and adriamycin (ADR), with or without low concentration FX11 (1 μM) (*n* = 3–6). ∗*P* < 0.05, ∗∗*P* < 0.01, ∗∗∗*P* < 0.001, and ∗∗∗∗*P* < 0.0001. LDHA, lactate dehydrogenase A; MCD, minimal change disease; TNF-α, tumor necrosis factor-alpha.

Podocyte injury was assessed post-LDHA inhibition to explore whether LDHA inhibition caused podocyte injury. LDHA inhibition was performed using the LDHA inhibitor FX11 and LDHA siRNA, and a reduction in LDHA activity was observed (Supplementary Figures S4A and B). FX11 showed no podocyte injury at low concentrations but was harmful at high levels (Figure 5a). LDHA inhibition by siRNA caused significant apoptosis (Figure 5b). Serum-treated podocytes from patients with MCD were further injured, even when low FX11 concentrations were administered (Figure 5c). Similarly, under tumor necrosis factor–α and adriamycin stimulation, low FX11 concentrations enhanced podocyte injury (Figures 5d and e). Overall, inhibiting anaerobic glycolysis had no significant effect under normal conditions; however, a slight decrease in anaerobic glycolysis exacerbated podocyte injury under injured conditions. These results suggested that injured podocytes depend more on the anaerobic glycolytic system, including LDHA.

### Sera From Patients With FSGS or LDHA Inhibition Reduced ACTN4 Expression and Promoted the Actin Cytoskeleton Rearrangement

**Figure 6:**
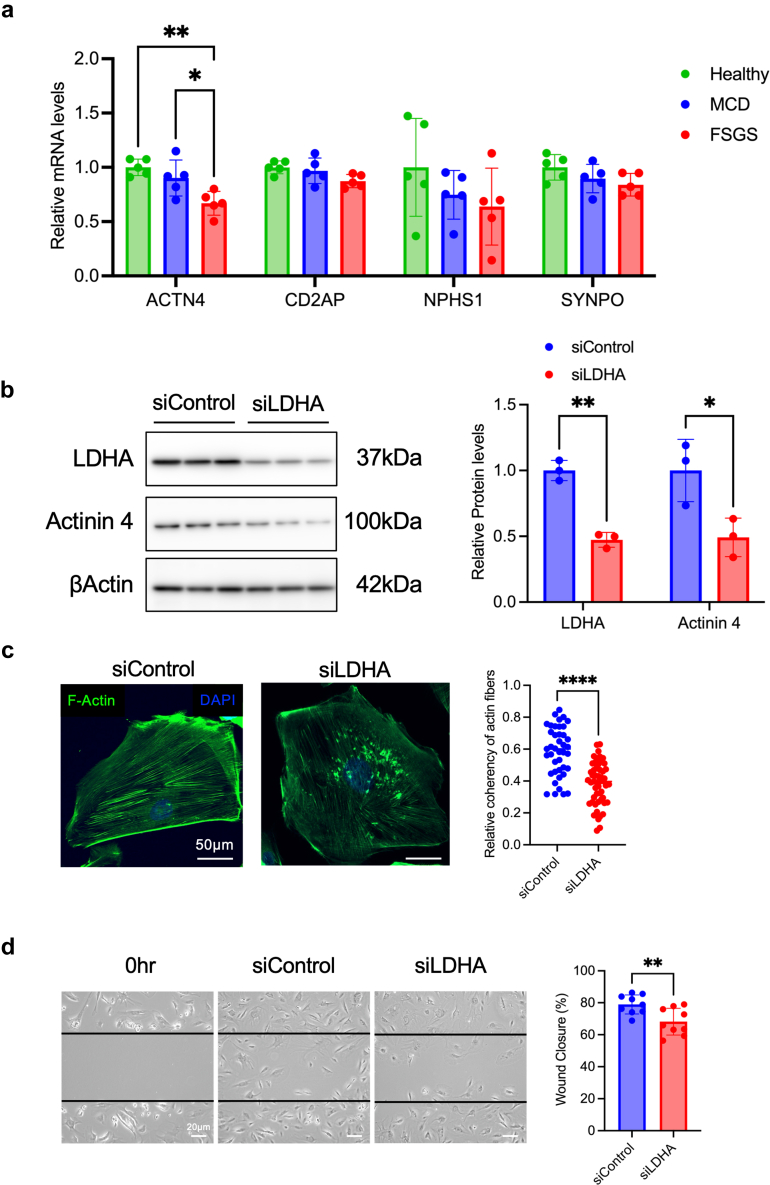
Sera from patients with FSGS or LDHA silencing affects α-actinin-4 (ACTN4) expression, leading to actin damage in podocytes. (a) Podocyte-associated genes (*ACTN4*, *CD2AP*, *NPHS2*, and *SYNPO*) in serum-treated podocytes from healthy individuals and patients with MCD or FSGS (*n* = 5 each). ∗*P* < 0.05. (b) Representative western blot analysis of LDHA and ACTN4 in cultured podocytes with or without LDHA silencing (*n* = 3). (c) Representative images of F-actin staining and coherency of actin fibers in cultured podocytes with or without LDHA silencing (*n* = 5). ∗∗∗∗*P* < 0.0001. The scale bar equals 50 μm. (d) Representative images of the podocyte migration and quantification of wound closure in cultured podocytes with or without LDHA silencing (*n* = 9). ∗∗*P* < 0.01. The scale bar equals 20 μm. ; FSGS, focal segmental glomerulosclerosis; LDHA, lactate dehydrogenase A; MCD, minimal change disease.

Podocyte-specific marker expression in podocytes treated with healthy and patient sera was evaluated using quantitative real time–polymerase chain reaction. ACTN4 expression was lower in serum-treated podocytes from patients with FSGS than in those from healthy individuals and patients with MCD (Figure 6a). Reducing LDHA using siRNA also reduced ACTN4 expression, coherence of actin stress fibers, and migration of podocytes (Figure 6b–d). Its activity inhibition with FX11 also resulted in comparable effects (Supplementary Figure S5A–C).

### Ldha-Deficient Mice Have More Segmental Sclerosis in Adriamycin-Induced Nephropathy

**Figure 7:**
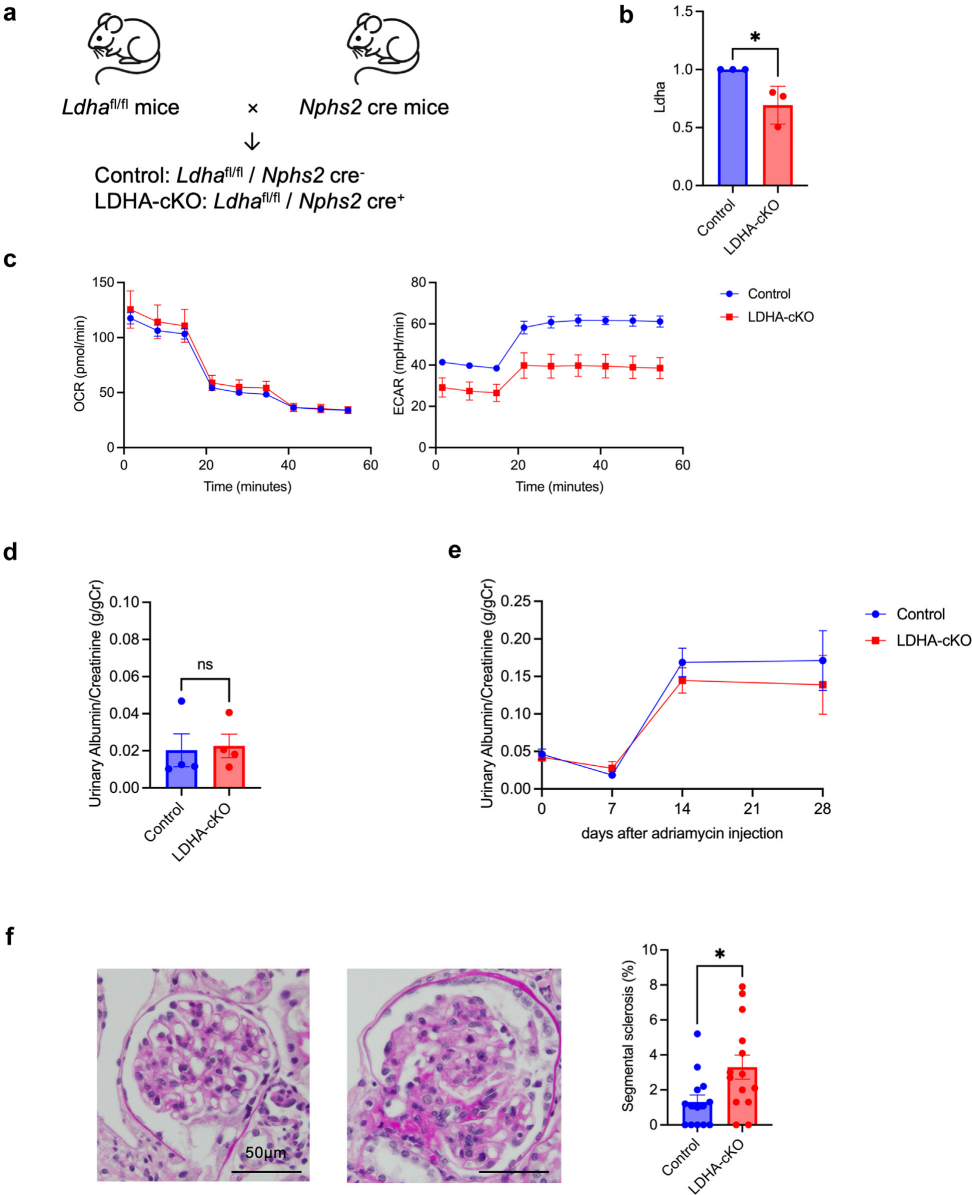
*Ldha* deletion in podocytes accelerates segmental sclerosis in podocyte injury. (a) Schema illustrating generation of podocyte-specific LDHA-cKO mice. (b) *Ldha* messenger RNA expression in isolated podocytes from control and podocyte-specific LDHA-cKO mice. (c) Real-time ATP rate assay results of isolated podocytes from control and podocyte-specific LDHA-cKO mice. (d) Urinary protein at 8 weeks of age in control and LDHA-cKO mice (*n* = 4). (e) Urinary protein in adriamycin-induced nephropathy (*n* = 14). (f) Representative pathologic images of adriamycin-induced nephropathy and the results of segmental sclerosis (*n* = 14). The scale bar equals 50 μm. ATP, adenosine triphosphate; LDHA-cKO, lactate dehydrogenase A-conditional knockout. L

We crossbred *Ldha*-flox mice with *NPHS2-*cre mice to create the first podocyte-specific *Ldha*-deficient mice (*Ldha*^fl/fl^/Cre^+^; Figure 7a). The *Ldha*^fl/fl^/Cre^+^ mice showed decreased *Ldha* in podocytes using quantitative real-time–polymerase chain reaction and decreased glycolysis in the real-time ATP rate assay (Figures 7b and c). Under normal conditions, *Ldha*^fl/fl^/Cre^+^ mice had no increase in urinary protein compared with *Ldha*^fl/fl^/Cre^-^ mice. No difference was found in urinary protein levels between the 2 groups postadriamycin nephropathy induction (Figures 7d and e); however, *Ldha*^fl/fl^/Cre^+^ mice had increased glomerular segmental sclerosis compared with *Ldha*^fl/fl^/Cre^-^ mice (Figure 7f). Segmental sclerosis incidence increases when the number of podocytes per glomerulus falls below 4 to 5.^22^^,^^23^ No significant difference was found in the number of podocytes in the glomeruli between *Ldha*^fl/fl^/Cre^+^ and *Ldha*^fl/fl^/Cre^−^ mice (Supplementary Figure S6A); however, when comparing the percentage of glomeruli with <4 podocytes to the total number of glomeruli, the rate was significantly higher in *Ldha*^fl/fl^/Cre^+^ mice than in *Ldha*^fl/fl^/Cre− mice (Supplementary Figure S6B).

## Discussion

We demonstrated that the severity of podocyte injury induced by the sera from patients with MCD or FSGS may help identify corticosteroid-resistant FSGS and that anaerobic glycolysis may be linked to FSGS progression. Sera from patients with FSGS and SRNS significantly induced podocyte cell death, which correlated with corticosteroid resistance and a high incidence of segmental lesions that are poor pathological prognostic factors. Using this *in vitro* system, we found that anaerobic glycolytic ATP production in podocytes was impaired in patients with corticosteroid-resistant FSGS with severe serum-induced injury. Decreased LDHA levels resulted in increased podocyte cell death and disrupted the actin cytoskeleton. FSGS lesions progressed in podocyte-specific *Ldha-*deficient mice with adriamycin-induced nephropathy. These findings suggest that anaerobic glycolysis dysregulation plays a key role in FSGS progression.

Recently, antinephrin antibodies in serum and diffuse punctate IgG staining in glomeruli were shown to be present in MCD or FSGS, particularly in the corticosteroid-dependent population with a good response to therapy.^24^ Validation studies have shown that antinephrin autoantibodies are widely present in adult patients with MCD and pediatric patients with idiopathic nephrotic syndrome.^25^ However, a definitive circulating factor has not been identified in FSGS.^2^^,^^3^ Serum or plasma from patients with primary FSGS increases protein permeability in a system that mimics the glomerular filtration barrier using isolated glomeruli or podocyte cell lines and induces proteinuria in rats.^26^, ^27^, ^28^ We examined podocyte injury using sera from patients with pretreatment and posttreatment FSGS. The sera from patients with posttreatment FSGS versus pretreatment FSGS showed a decrease in podocyte injury. These findings emphasize that the sera from patients with FSGS can cause podocyte injury. We examined the relationship between podocyte injury caused by sera from patients with MCD or FSGS and clinical parameters. A recent study showed diffuse microvillous transformation on electron microscopy as a finding indicative of therapeutic response, whereas total and segmental sclerosis, interstitial fibrosis and tubular atrophy, and segmental obliteration were poor prognostic indicators.^29^ Here, the percentage of annexin V–positive cell death induced by the sera from patients with primary FSGS was not associated with diffuse punctate IgG staining or microvillous transformation (Supplementary Figure S7A and B). However, the degree of cell death was significantly associated with interstitial fibrosis and tubular atrophy and segmental obliteration—both poor prognostic pathological findings—and the degree of clinical resistance to corticosteroids. These results suggest that this *in vitro* system is suitable for identifying treatment-resistant MCD or FSGS cases, although validation studies involving other nephrotic diseases are needed.

The kidneys consume significant energy to maintain body homeostasis, primarily utilizing glucose, fatty acids, amino acids, and ketone bodies; however, renal resident cells have various metabolic pathways that are preferentially used by different nephron segments.^7^^,^^30^^,^^31^ Ozawa *et al.*^10^ reported that glycolysis contributes to ATP production in the cortical part of podocytes, whereas Chen *et al.*^32^ showed that mitochondria are mainly distributed around the nucleus and glycolytic enzymes, including pyruvate kinase M2 and LDHA are predominantly distributed in the foot processes. Here, LDHA inhibition induced podocyte cell death and cytoskeletal remodeling in concentration-dependently, indicating that anaerobic glycolysis is an important energy source for podocytes under physiological conditions. However, the podocyte-specific *Ldha-*deficient mice did not exhibit proteinuria despite significantly impaired ATP production from glycolysis, suggesting that reduced anaerobic glycolysis has no significant effect on podocyte homeostasis under healthy conditions. Glomerular component cells produce less ATP than other nephron segments under normal conditions.^7^ Brinkkoetter *et al.*^12^ reported minimal effects of impaired mitochondrial dynamics, biosynthesis, or mitochondrial DNA transcription on podocyte injury and renal function. This suggests that homeostasis can be maintained by supplementing ATP production from other sources at a steady state, even with reduced glycolysis or mitochondria-derived ATP production.

Recent studies have associated the development of renal diseases with abnormal energy metabolism.^30^ In diabetic kidney disease, podocytes exhibit impaired glycolysis; however, pyruvate kinase M2 activation in podocytes restores mitochondrial dysfunction and prevents diabetic kidney disease development.^33^ The activation of the renin-angiotensin system, such as angiotensin II, which is common in diabetic kidney disease and hypertensive nephropathy, is involved in renal damage progression, and associated with reduced pyruvate kinase M2 function.^32^ However, podocyte injury leading to FSGS lesions is found in patients with mitochondria-related genetic mutations. Although increased reactive oxygen species production can also injure podocytes in these cases, whether this can be explained by decreased mitochondrial ATP production remains unclear.^34^ Sidhom *et al.*^35^ revealed a mitochondrial electron transfer system–independent role of coenzyme Q in podocyte damage in coenzyme Q-deficient mice. We found that podocyte cell death induced by the sera from patients with FSGS was associated with a reduction in glycolysis-derived ATP production but not with mitochondria-derived ATP production. The relationship between glycolytic capacity and apoptosis indicates that a decrease in maximum glycolytic activity worsens cell damage during podocyte injury. These findings suggest that a reduced anaerobic glycolysis, rather than a decreased total ATP production, may hinder cell survival during podocyte injury. In fact, low concentrations of LDHA inhibitors significantly induced apoptosis during injury. No difference was found in proteinuria; however, FSGS lesions were significantly more pronounced in *Ldha*^fl/fl^ /Cre^+^ mice than in *Ldha*^fl/fl^ /Cre^−^ mice upon injury with adriamycin. This may be because the high rate of advanced podocyte loss in the glomerulus led to a significant increase in segmental glomerulosclerosis. LDHA downregulation may result in insufficient energy storage to manage stress injury in podocytes, indicating that anaerobic glycolysis is important for podocyte homeostasis during injury. Furthermore, circulating factors in the sera from patients with FSGS may disrupt the anaerobic glycolysis of podocytes.

Actin cytoskeleton regulation is associated with the formation and migration of podocyte foot processes, and approximately half of the intracellular ATP is required for its reorganization.^36^ LDHA inhibition significantly disrupted the actin cytoskeleton and reduced migration ability, particularly during injury; therefore, glycolysis is important for regulating actin cytoskeleton during injury.^10^ Although an insufficient energy supply activates autophagy to maintain podocytes’ survival, abnormal autophagy causes podocyte cytoskeleton remodeling.^37^, ^38^, ^39^ mTOR is an important regulator of autophagy induction, where an activated mTOR suppresses autophagy and a suppressed mTOR promotes autophagy. A lack of energy supply, which triggers actin cytoskeleton remodeling in podocytes, suppresses mTOR. However, in our study, decreased energy production because of LDHA inhibition did not affect phosphorylated mTOR expression in podocytes (Supplementary Figure S8). Such mTOR-induced autophagy dysregulation may further reduce glycolysis, consequently promoting actin cytoskeleton remodeling and forming a vicious cycle. ACTN4 is an actin-binding protein that cooperates with synaptopodin in podocytes to regulate actin fiber bundle activity.^40^ It is a causative gene of familial FSGS, and mutations or loss of ACTN4 promote podocyte detachment.^41^, ^42^, ^43^ Here, LDHA inhibition significantly suppressed ACTN4 expression, suggesting that podocyte detachment occurs in corticosteroid-resistant FSGS via reduced ACTN4 expression and decreased energy compensation due to reduced LDHA activity. Elevated ACTN4 expression in prostate cancer upregulates LDHA expression, and other metabolic effects mediated by changes in ACTN4 expression have been suggested.^44^

Finally, we discuss the limitations of the metabolome analysis conducted in this study. The orthogonal partial least squares discriminant analysis model demonstrated excellent explanatory power (R^2^Y = 0.782) and moderate predictive ability (Q^2^ = 0.461). However, the relatively large difference between R^2^Y and Q^2^ (0.321), with the limited sample size, suggests a potential risk of overfitting. Furthermore, because the data are human-derived, individual variability and background factors may have influenced the results. Future studies should focus on creating more robust, reproducible, and generalizable predictive models by increasing the sample size and validating the model with independent datasets. In conclusion, our study demonstrated that analyzing cell death in MCD or FSGS serum–treated human podocytes is a useful tool for identifying FSGS lesions and corticosteroid resistance and that anaerobic glycolysis disorders are associated with FSGS progression. These findings suggest that anaerobic glycolysis is a potential therapeutic target for podocytopathy.

## Disclosure

All the authors declared no competing interests.

## Supplementary Material

Supplementary File (PDF) **Figure S1.** Metabolome analysis of podocytes treated with sera from patients with MCD or FSGS. **Figure S2.** Podocyte injury inversely correlates with the glycolytic ATP production rate in podocytes treated with sera from patients with SRNS. **Figure S3.** Podocyte injury inversely correlates with glycolytic capacity in podocytes treated with sera from patients with FSGS. **Figure S4.** Lactate dehydrogenase A (LDHA) activity in podocytes under LDHA inhibition. **Figure S5.** FX11 damages actin structure in podocytes. **Figure S6.** Podocyte number and percentage of glomerulus with severe podocyte loss in control and LDHA-cKO mice with adriamycin-induced nephropathy. **Figure S7.** The relationship between pathological findings and the apoptosis rate of podocytes induced by sera from patients. **Figure S8.** LDHA inhibition does not affect phosphorylated mTOR expression.

## Footnotes

Supplementary File (PDF)
**Figure S1.** Metabolome analysis of podocytes treated with sera from patients with MCD or FSGS. **Figure S2.** Podocyte injury inversely correlates with the glycolytic ATP production rate in podocytes treated with sera from patients with SRNS. **Figure S3.** Podocyte injury inversely correlates with glycolytic capacity in podocytes treated with sera from patients with FSGS. **Figure S4.** Lactate dehydrogenase A (LDHA) activity in podocytes under LDHA inhibition. **Figure S5.** FX11 damages actin structure in podocytes. **Figure S6.** Podocyte number and percentage of glomerulus with severe podocyte loss in control and LDHA-cKO mice with adriamycin-induced nephropathy. **Figure S7.** The relationship between pathological findings and the apoptosis rate of podocytes induced by sera from patients. **Figure S8.** LDHA inhibition does not affect phosphorylated mTOR expression.
