# Supplementary material for: Dysregulated Anaerobic Glycolysis in Podocytes is Relevant to the Progression of Focal Segmental Glomerulosclerosis
Source: Kidney Int Rep. 2025 Jun 18;10(9):3239–54. doi: 10.1016/j.ekir.2025.06.022 (PMC12446983; doi:10.1016/j.ekir.2025.06.022)

SUPPLEMENTARY MATERIAL

Supplementary Figure S1. Metabolome analysis of podocytes treated with sera from patients with MCD or FSGS

(a) Principal component analysis of metabolites in podocytes treated with sera from MCD or FSGS patients (n=5 each). (b) One or more Variable Importance in Projection (VIP) score plots of metabolites in podocytes treated with sera from patients with MCD or FSGS.

MCD, minimal change disease; FSGS, focal segmental glomerulosclerosis

Supplementary Figure S2. Podocyte injury inversely correlates with the glycolytic ATP production rate in podocytes treated with sera from patients with SRNS

(a) Real-Time ATP Rate Assay of podocytes treated with sera from patients with SSNS (n=13) or SRNS (n=15) detected by extracellular flux analyzer. (b) Scatterplot showing the correlation between podocyte apoptosis and total/ mitochondrial/ glycolytic ATP production rate in SSNS (n=13) or SRNS (n=15).

ATP, adenosine triphosphate; SSNS, corticosteroid-sensitive nephrotic syndrome; SRNS, corticosteroid-resistant nephrotic syndrome

Supplementary Figure S3. Podocyte injury inversely correlates with glycolytic capacity in podocytes treated with sera from patients with FSGS

(a) Glycolysis Stress Test of podocytes treated with sera from patients with MCD (n=11) or FSGS (n=19) detected by extracellular flux analyzer. (b) Scatterplot showing the correlation between podocyte apoptosis and glycolysis, glycolytic capacity, or glycolytic reserve.

MCD, minimal change disease; FSGS, focal segmental glomerulosclerosis

Supplementary Figure S4. Lactate dehydrogenase A (LDHA) activity in podocytes under LDHA

inhibition

(a) LDHA activity of podocytes with or without FX11 (1–10 μM) (n=3 each). *$P < 0.05$, **$P < 0.01$.

(b) LDHA activity of podocytes with or without LDHA silencing (n=3 each). **$P < 0.01$.

Supplementary Figure S5. FX11 damages actin structure in podocytes

(a) Representative western blot analysis of α-actinin 4 (ACTN4) in cultured podocytes with or without FX11 (1–10 μM) (n=3). *$P < 0.05$, **$P < 0.01$. (b) Representative images of F-actin staining and coherency of actin fibers of cultured podocytes with or without FX11 (1–10 μM) (n=5). **$P < 0.01$, ***$P < 0.001$. The scale bar equals 20 μm. (c) Representative images of the podocyte migration and quantification of wound closure of cultured podocytes with or without FX11 (1–10 μM) (n=9). ***$P < 0.001$, ****$P < 0.0001$. The scale bar equals 20 μm.

Supplementary Figure S6. Podocyte number and percentage of glomerulus with severe podocyte loss in control and LDHA-cKO mice with adriamycin nephropathy

(a) Podocyte number in control and LDHA-cKO mice with adriamycin-induced nephropathy. (b) Percentage of glomeruli with four or fewer podocyte numbers per total glomerulus in control and LDHA-cKO mice with adriamycin-induced nephropathy. *P < 0.05

Supplementary Figure S7. The relationship between pathological findings and the apoptosis rate of podocytes induced by sera from patients

(a, b) Scatterplot showing the correlation between podocyte apoptosis and diffuse punctate IgG staining (a), Microvillous transformation (b).

Supplementary Figure S8. LDHA inhibition does not affect phosphorylated mTOR expression

Representative Western blot analysis of phospho-mTOR, mTOR, and LDHA expression in cultured podocytes by LDHA siRNA (n=3).

mTOR, mechanistic target of rapamycin; LDHA, lactate dehydrogenase A

Supplementary Figure S1

a

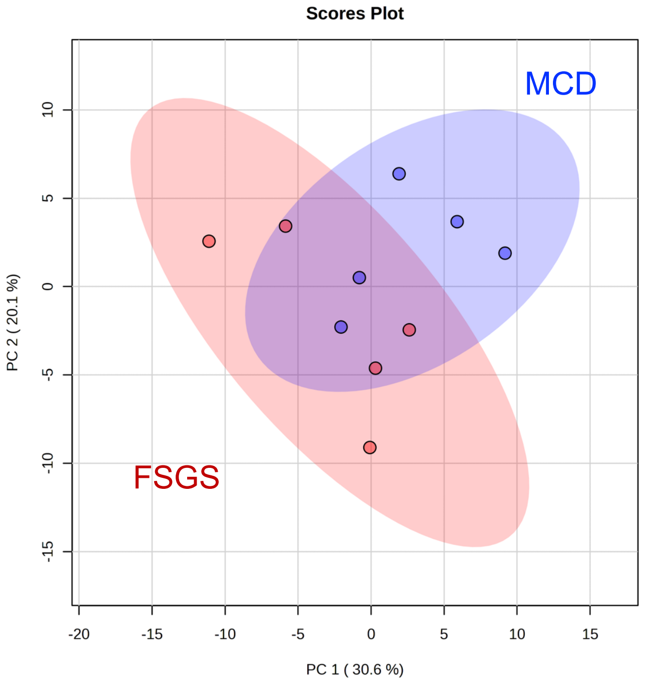

b

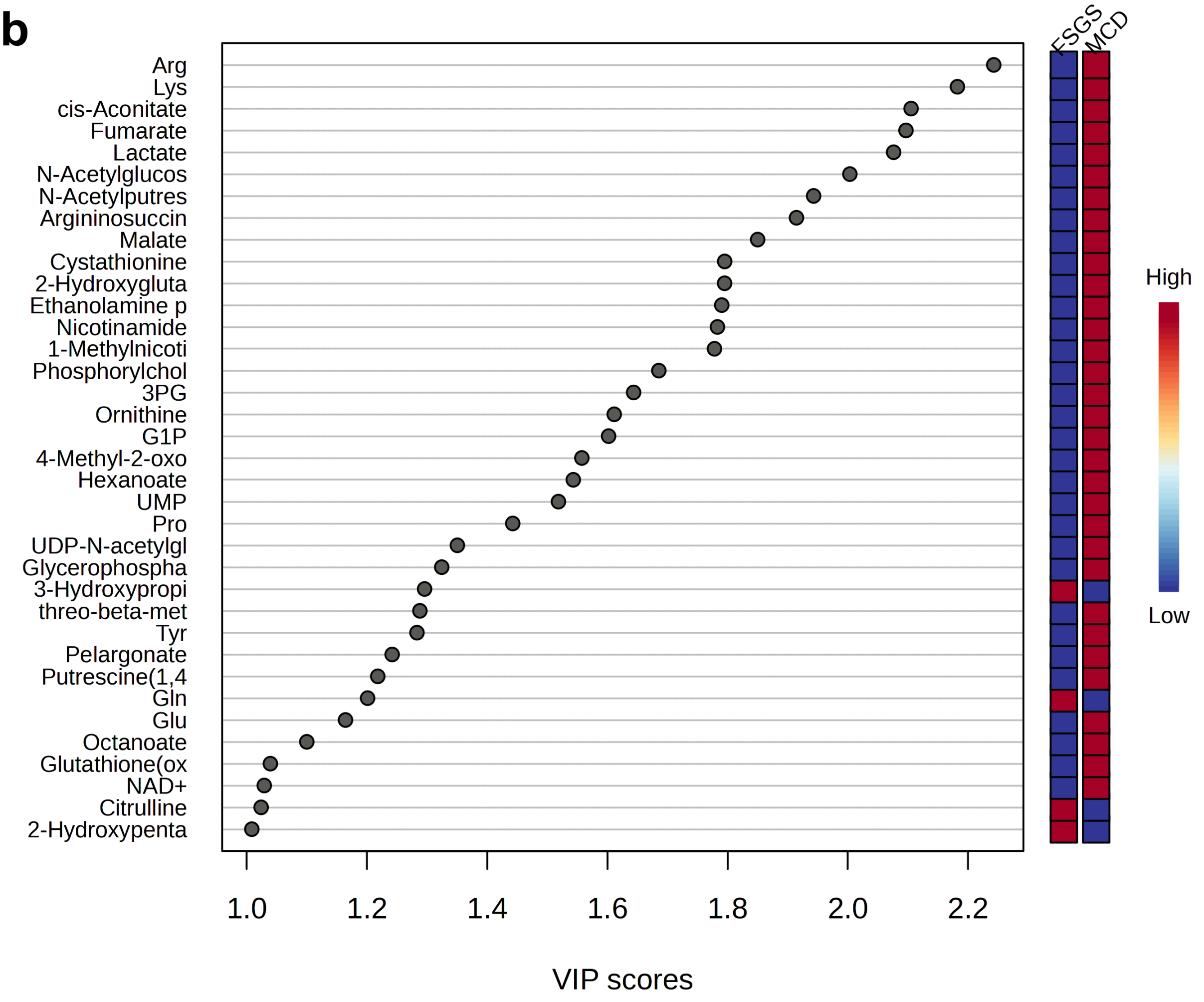

Supplementary Figure S2

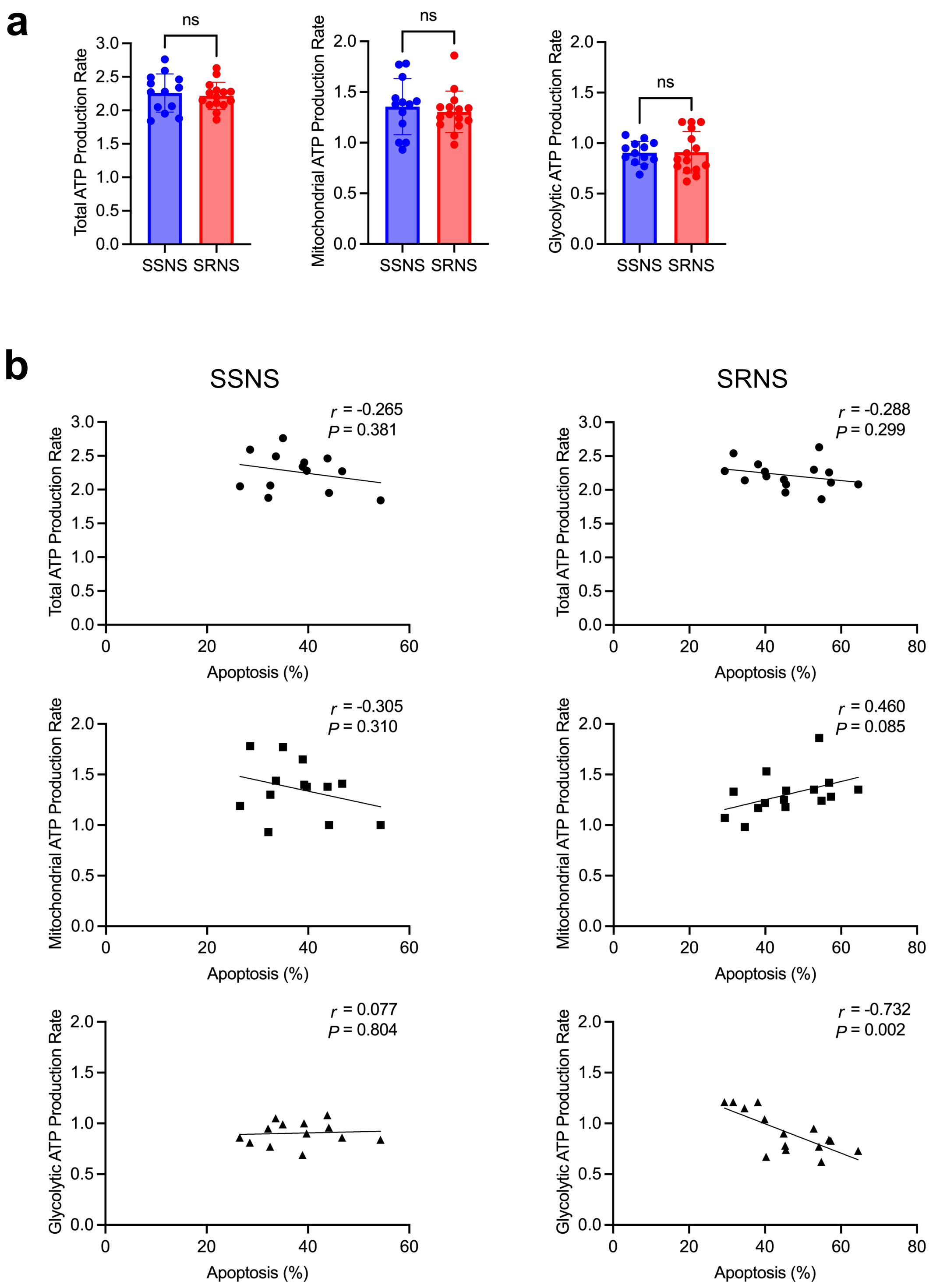

Supplementary Figure S3

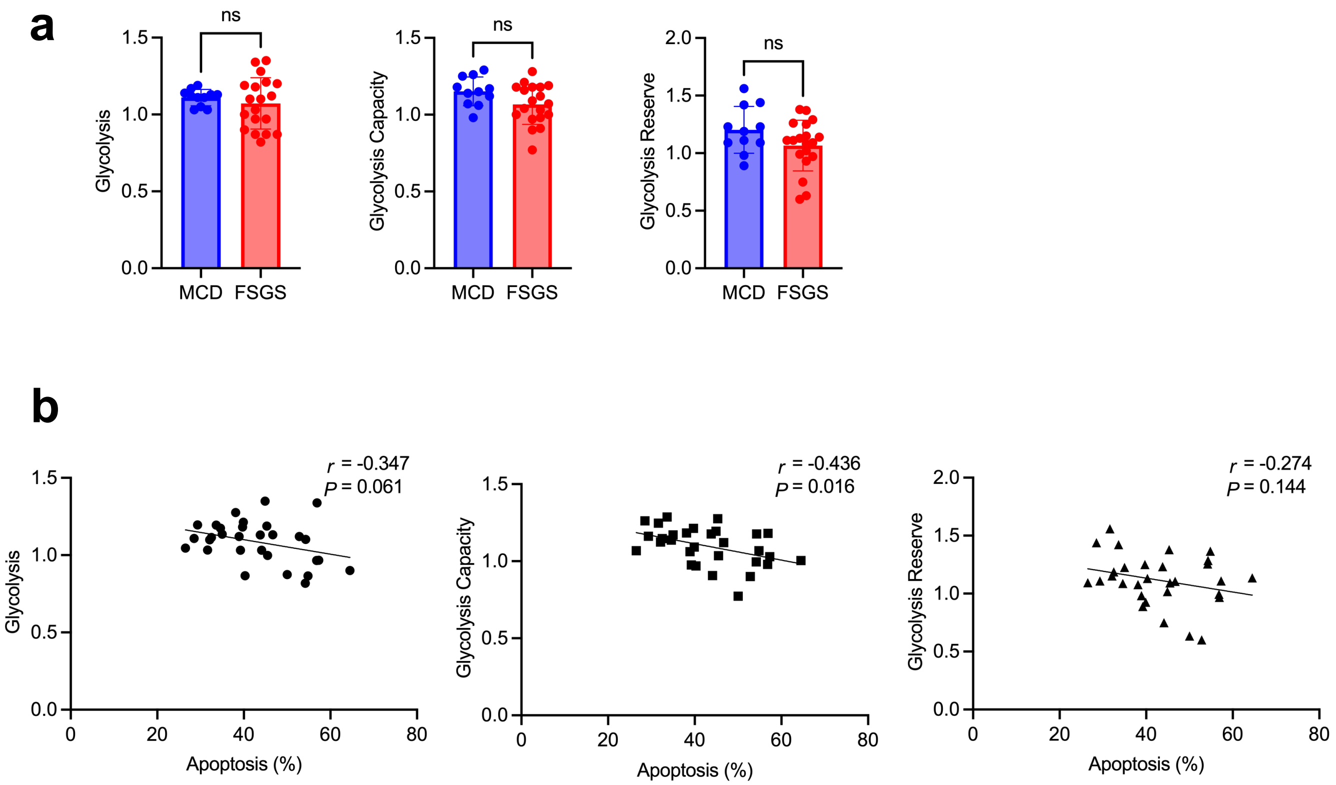

Supplementary Figure S4

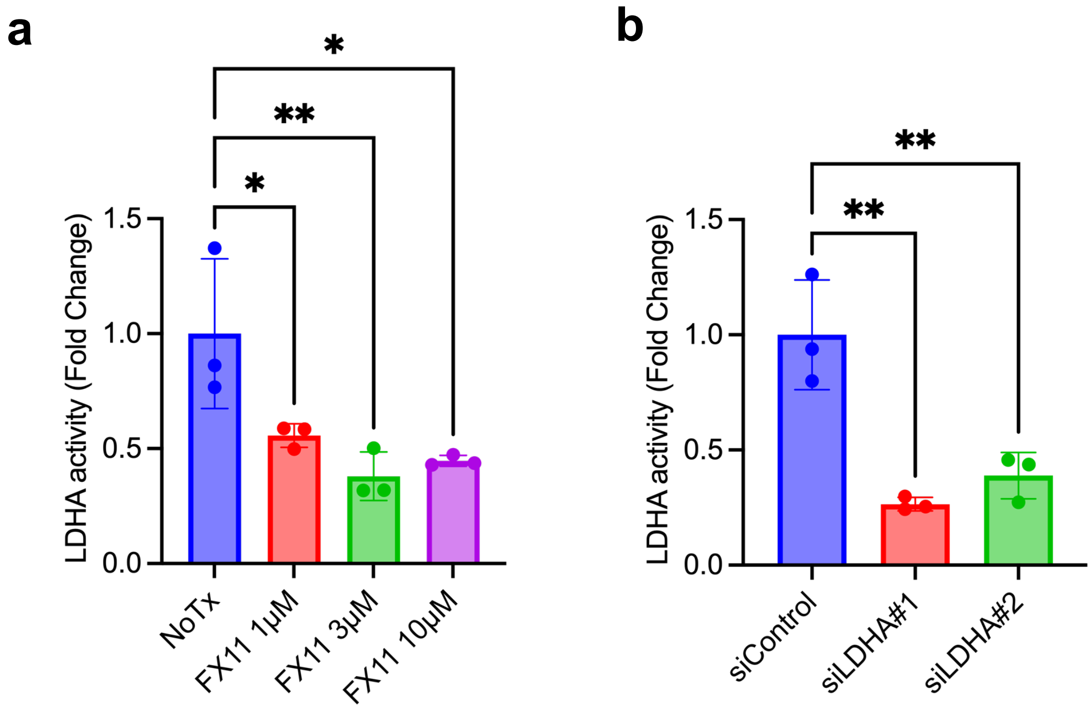

Supplementary Figure S5

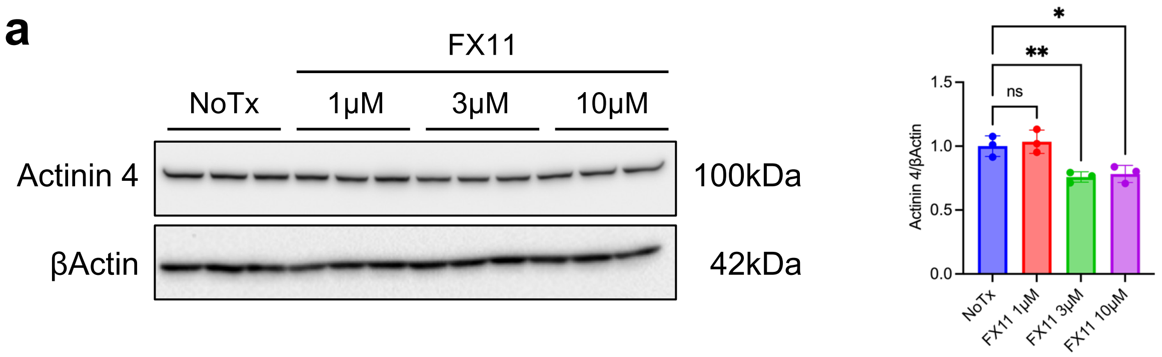

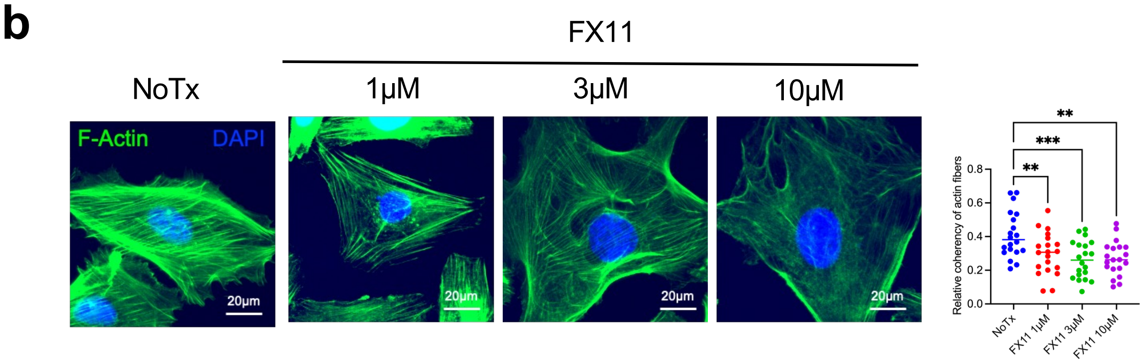

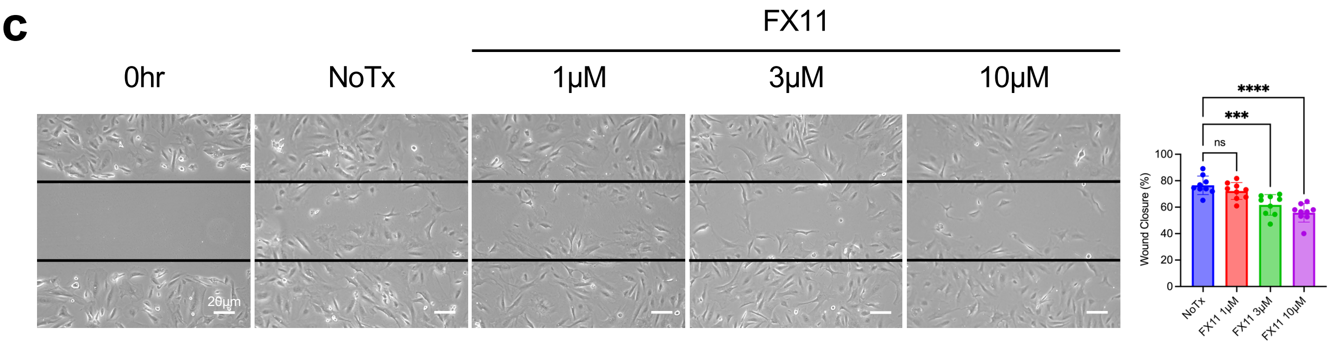

Supplementary Figure S6

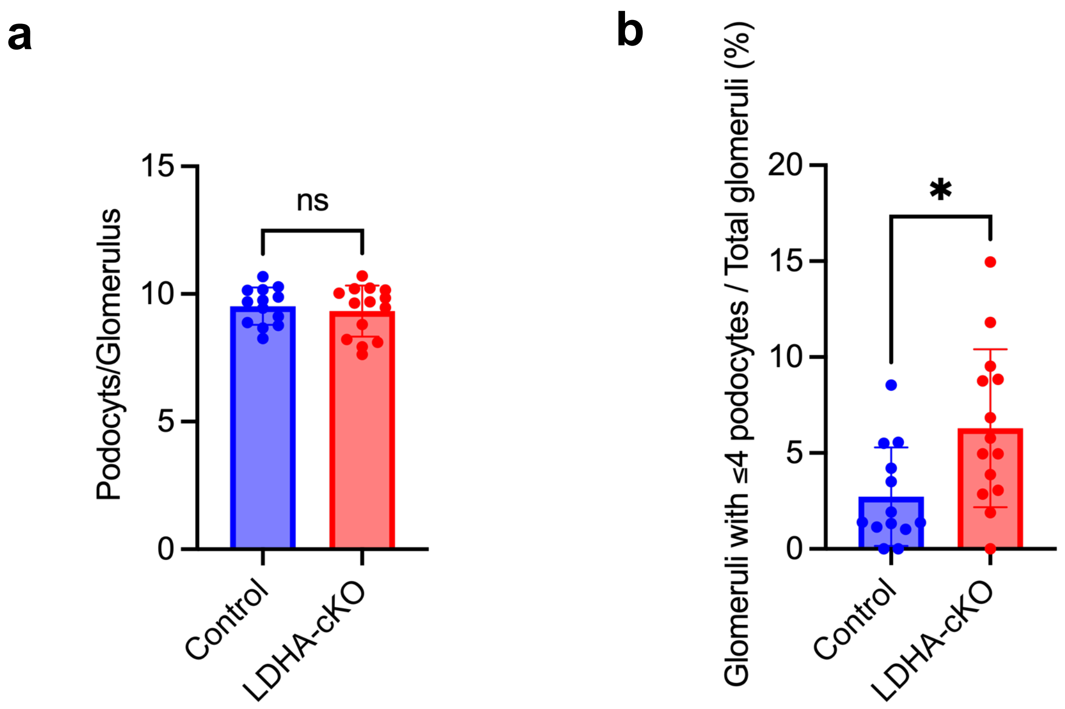

Supplementary Figure S7

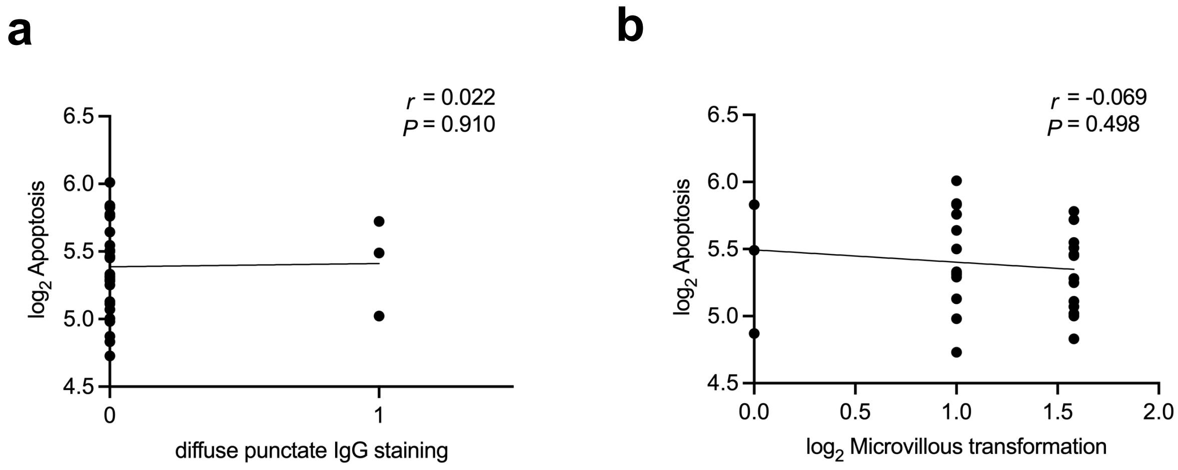

Supplementary Figure S8

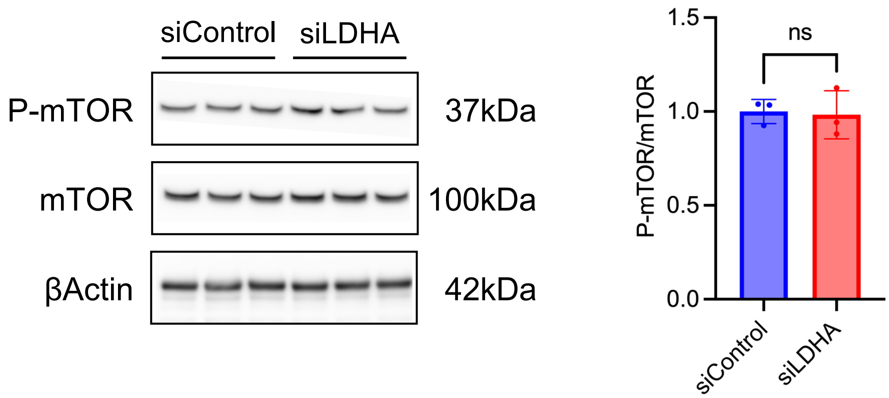

Supplement: Supplementary File (PDF) — Figure S1. Metabolome analysis of podocytes treated with sera from patients with MCD or FSGS. Figure S2. Podocyte injury inversely correlates with the glycolytic ATP production rate in podocytes treated with sera from patients with SRNS. Figure S3. Podocyte injury inversely correlates with glycolytic capacity in podocytes treated with sera from patients with FSGS. Figure S4. Lactate dehydrogenase A (LDHA) activity in podocytes under LDHA inhibition. Figure S5. FX11 damages actin structure in podocytes. Figure S6. Podocyte number and percentage of glomerulus with severe podocyte loss in control and LDHA-cKO mice with adriamycin-induced nephropathy. Figure S7. The relationship between pathological findings and the apoptosis rate of podocytes induced by sera from patients. Figure S8. LDHA inhibition does not affect phosphorylated mTOR expression. [file mmc1.pdf]
